# Electrocardiogram Risk Score and Prevalence of Subclinical Atherosclerosis: A Cross-Sectional Study

**DOI:** 10.3390/jpm12030463

**Published:** 2022-03-14

**Authors:** Minji Kang, Yoosoo Chang, Jeonggyu Kang, Yejin Kim, Seungho Ryu

**Affiliations:** 1 Center for Cohort Studies, Total Healthcare Center, Kangbuk Samsung Hospital, School of Medicine, Sungkyunkwan University, Seoul 04514, Korea; minjikang53@gmail.com (M.K.); kamgjk@gmail.com (J.K.); reenya273@gmail.com (Y.K.); 2 Department of Occupational and Environmental Medicine, Kangbuk Samsung Hospital, School of Medicine, Sungkyunkwan University, Seoul 03181, Korea; 3 Department of Clinical Research Design & Evaluation, SAIHST, Sungkyunkwan University, Seoul 06355, Korea

**Keywords:** ECG risk score, atherosclerosis, coronary artery calcium, low-risk population, young adults

## Abstract

Integrated abnormal electrocardiogram (ECG) parameters predict the risk of cardiovascular disease (CVD); however, its relationship with subclinical CVD is unknown. We aimed to evaluate the association between the integrated ECG risk score and the prevalence of coronary artery calcium (CAC). A cross-sectional study comprised 134,802 participants with no known CVD who underwent ECG and CAC computed tomography. The ECG risk score was the sum of five ECG abnormalities: heart rate of >80 beats, QRS of >110 ms, left ventricular hypertrophy, T-wave inversion, and prolonged QTc. A multinomial regression model was used to estimate the prevalence ratios (PRs) and their 95% confidence intervals (CIs) for prevalent CAC. The prevalence of CAC progressively increased as the ECG risk score increased. After adjustment for conventional CVD risk factors and other confounders, the multivariable-adjusted PRs (95% CI) for a CAC of 1–100 in the 1, 2, and ≥3 ECG risk score groups were 1.06 (1.02–1.10), 1.12 (1.03–1.22), and 1.19 (1.00–1.42), respectively, while the corresponding PRs for a CAC of >100 were 1.03 (0.95–1.12), 1.44 (1.25–1.66), and 1.75 (1.33–2.29), respectively. Integrative ECG scoring may help identify individuals requiring lipid-lowering medications, even in young and asymptomatic populations.

## 1. Introduction

Cardiovascular disease (CVD) is the leading cause of death, accounting for 31% of deaths worldwide [1]. The risk stratification for CVD is primarily focused on high-risk populations [2,3]. The CVD burden in adults aged >50 years has been steadily decreasing, while it has been increasing among the young population over the past decades [4,5]. Therefore, recent studies have highlighted the importance of enhancing prevention strategies for young and low-risk groups. Improving the tools used for CVD risk stratification and the early detection of CVD risk among young and low-risk populations would be necessary to reduce the global burden of CVD.

Electrocardiogram (ECG) is a non-invasive and widely used method for evaluating underlying heart conditions and is helpful in detecting ischemic heart diseases [6]. However, ECG abnormalities related to coronary artery disease can vary, and a single ECG abnormality is insufficient when used alone to predict coronary artery disease [7,8]. Additionally, many individuals with subclinical atherosclerosis have no definite major abnormalities in ECG findings; thus, we hypothesized that the integrated ECG score would better reflect heart conditions than a single abnormal signal possibly combining several features [9]. Indeed, several ECG parameters such as increased heart rate and markers of abnormal cardiac depolarization and repolarization were previously associated with an increased risk of subclinical atherosclerosis [10,11,12]. However, it is unclear whether integrating multiple ECG abnormalities into a risk score would better identify patients at a high risk of atherosclerotic burden than a single variable. Moreover, it remains controversial whether ECG is effective in stratifying CAD risks and is suitable as a screening tool for low-risk populations [13,14].

Coronary artery calcification (CAC), measured using computed tomography (CT), is a useful tool for detecting subclinical atherosclerosis [15]. It classifies intermediate-risk populations who might benefit from cholesterol-lowering agents [16]. Detecting the preclinical stage of CVD is crucial to prevent the progression of CVD since it enables early intervention and treatment [17]. Several studies evaluated the relationship between individual ECG parameters and CAC [18,19]. To the best of our knowledge, no study has demonstrated an association between integrated ECG parameters and the prevalence of CAC scores. Therefore, we aimed to evaluate whether the integrated ECG risk score is independently associated with subclinical CAC.

## 2. Materials and Methods

### 2.1. Study Population

The Kangbuk Samsung Health Study is a cohort study of South Korean men and women aged 18 years and older who underwent comprehensive annual or biennial examinations at Kangbuk Samsung Hospital Total Healthcare Center in Seoul and Suwon, Korea [20]. The majority of participants were employees of companies and local governmental organizations and their spouses. Our study population consisted of Kangbuk Samsung Health Study participants who underwent multi-detector CT (MDCT) for the estimation of CAC as part of the annual health examination from 2011 to 2018.

**Figure 1:**
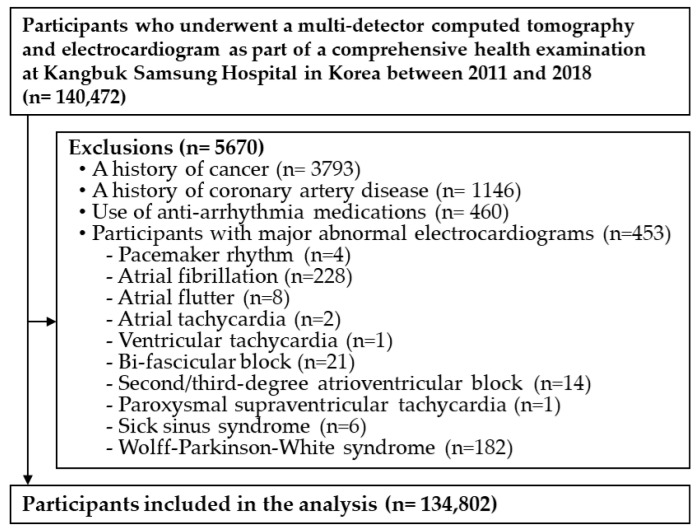
Flow chart describing the selection of the study participants.

We excluded participants who had missing data on ECG or CAC as well as those who met one or more of the exclusion criteria (history of cancer, history of CAD, and use of anti-arrhythmia medications); participants with major abnormal ECGs including pacemaker rhythm, atrial fibrillation, atrial flutter, atrial tachycardia, ventricular tachycardia, bifascicular block, second/third-degree atrioventricular block, paroxysmal supraventricular tachycardia, sick sinus syndrome, and Wolff–Parkinson–White syndrome were also excluded. The total number of eligible participants was 134,802 (Figure 1).

This study was approved by the Institutional Review Board of Kangbuk Samsung Hospital (IRB No. KBSMC 2020-12-016) and was conducted in accordance with the Declaration of Helsinki. The requirement for informed consent was waived owing to the use of a pre-existing de-identified dataset that was routinely collected during the health screening process.

### 2.2. Measurement

A health questionnaire as a part of health examination consisted of questions related to the medical history, family history, medication history, smoking status, alcohol consumption, physical activity, and educational level of the participants. Smoking status was categorized as never, former, and current smokers. Average alcohol intake was categorized as alcohol intake of ≥20 g/day in women and alcohol intake of ≥30 g/day in men [21]. Physical activity was categorized as regular exercise three or more times a week and exercise less than three times a week. Education level was categorized as less than college graduate or higher.

Hypertension was defined as having a systolic blood pressure of equal to or more than 140, diastolic blood pressure of equal to or more than 90, having a history of hypertension, or taking anti-hypertensive medications. Weight and height were measured by trained staff as part of the annual health examination. Body mass index (BMI) was calculated as body weight (kg) divided by height in meter squared (m^2^). The serum measurements included fasting serum glucose, hemoglobin A1c (HbA1c), total cholesterol, low-density lipoprotein cholesterol (LDL-C), high-density lipoprotein cholesterol (HDL-C), triglyceride level, creatinine, and high-sensitivity C-reactive protein (hsCRP). Diabetes was defined as having a fasting serum glucose level of equal to or more than 126, HbA1c level of equal to or more than 6.5, having a history of diabetes, or taking anti-diabetic medications. The estimated glomerular filtration rate (eGFR) was calculated using the Chronic Kidney Disease Epidemiology Collaboration equation [22].

### 2.3. Measurement of Electrocardiogram

A 12-lead ECG was recorded at a 25 mm/s paper and at 1 mV/cm amplification with an ECG recorder (CardiMax FX-7542; Fukuda Denshi., Ltd., Tokyo, Japan). HR (beats per minute), PR interval duration (ms), QRS duration (ms), R and S wave amplitude (mV), T wave amplitude (mV), and QT interval duration (ms) were determined using ECG analysis software (PI-19E, Fukuda Denshi). The ECG was interpreted and codified using the Minnesota Code [23] by two experienced cardiologists, and cases with arrhythmia and conduction disturbances belonging to exclusion criteria were dropped in the analysis. We included the following minor ECG abnormalities, which are known to be associated with heart disease [9,24]: HR of >80 beats, PR interval of >220 ms, QRS of >110 ms, left ventricular hypertrophy (LVH) determined using the Sokolow–Lyon criterion, T-wave inversion, and prolonged QTc (>450 ms in men; >460 ms in women) determined using the Bazett formula. T-wave inversion corresponded to the criteria of Minnesota codes 5–1 and 5–2, defined as greater than 1 mm of negative T-wave amplitude in the anterior or lateral leads (V2–6, I, aVL, II) with R amplitude > 5.0 mm or in the inferior leads (II and aVF) with mainly upright QRS complexes [23]. We categorized the study population into 0 ECG risk score, 1 ECG risk score, 2 ECG risk score, or ≥3 ECG risk scores based on the total number of abnormal ECG parameters.

### 2.4. Measurement of CAC by Multidetector CT

Cardiac MDCT was performed using a LightSpeed VCT XTe-64 slice MDCT scanner (GE Healthcare, Tokyo, Japan) with the following standard scanning protocol: 2.5 mm thickness, 400 ms rotation time, 120 kV voltage, and 124 mAS (310 mA * 0.4 s) tube under ECG-gated dose modulation [25]. Calcification scores were determined using the Agatston method [26]. The CAC score was categorized as 0, 1–100, and >100, in which cholesterol-lowering intervention is recommended to prevent future cardiovascular events [16,17].

### 2.5. Statistical Analyses

The characteristics of the study participants were presented according to the ECG risk score category. Since there were large differences in age and sex between ECG score categories, all characteristics were presented as age- and sex-adjusted mean or proportion with 95% confidence intervals (CIs).

We evaluated the association of individual ECG parameters with the prevalence of CAC to determine which individual ECG parameters should be included in the ECG risk scoring [24] and the estimated prevalence ratios (PRs) with 95% CIs for prevalent CAC of >0 using a Poisson regression with robust variance. First, each individual ECG parameter was adjusted for age and sex, and the prevalence of CAC was determined. Then, ECG parameters were analyzed in the same model simultaneously to demonstrate the independent association of each ECG parameter with the prevalence of CAC.

Multinomial logistic regression was used to estimate the PRs and 95% CIs for prevalent CAC according to the ECG risk score category. We initially constructed an age- and sex-adjusted model and then a multivariate-adjusted model with further adjustment for center, year of screening, BMI, smoking status, alcohol intake, physical activity, educational level, family history of heart disease, history of diabetes, history of hypertension, LDL-C, HDL-C, and triglyceride level.

Additionally, a Tobit regression model, an analytic model that fits the distribution of CAC, for the natural log (CAC score + 1) with the Huber–White estimation of standard errors was used to estimate the CAC ratio and 95% CI to demonstrate the association between ECG risk score and CAC score as a continuous variable [27]. Stata version 16.1 (StataCorp LP, College Station, TX, USA) was used to perform all statistical analyses; a *p*-value of < 0.05 was considered significant.

## 3. Results

The baseline characteristics of the study population based on the ECG risk scores are shown in Table 1. The mean age of the 0 ECG risk score group was 41.3 (41.2–41.3) years, while that of the ≥3 risk score group was 44.2 (43.7–44.7) years. Participants with higher ECG risk scores were likely to be older, male, with a history of HTN, history of diabetes, higher BMI, excess alcohol intake, higher total cholesterol level, and less likely to be current smokers, perform regular exercise, and have a high education level.

Table 2 presents the associations of each abnormal ECG parameter with the prevalence of CAC. In the age- and sex-adjusted model, HR of >80 bpm, QRS of >110 ms, LVH, T-wave inversion, and prolonged QTc were significantly associated with prevalent CAC, but PR interval > 220 ms was not. In the mutually adjusted model, five ECG abnormalities remained significant and were therefore included in the ECG risk score for further analysis.

Table 3 demonstrates the PRs for CAC in each ECG risk score group. In the multivariate-adjusted model, the risk of prevalent CAC progressively increased as the ECG risk score increased. The multivariate-adjusted PRs (95% CI) for a CAC of >0–100 in the 1, 2, and ≥3 ECG risk score groups were 1.06 (1.02–1.10), 1.12 (1.03–1.22), and 1.19 (1.00–1.42), respectively, while the corresponding PRs (95% CI) for a CAC of >100 were 1.03 (0.95–1.12), 1.44 (1.25–1.66), and 1.75 (1.33–2.29), respectively. After further adjustment for eGFR quintile and hsCRP quintile, the associations were slightly attenuated but remained significant; corresponding PRs (95% CI) for a CAC of >100 in the 1, 2, and ≥3 ECG risk score groups were 0.98 (0.89–1.09), 1.55 (1.32–1.82), and 1.61 (1.16–2.23), respectively.

In the Tobit regression analyses (Table 4), the age- and sex-adjusted CAC score ratios of the 1, 2, and ≥3 ECG risk score groups were 1.03 (1.02–1.04), 1.22 (1.20–1.34), and 1.21 (1.18–1.25), respectively, while the corresponding multivariate-adjusted CAC score ratios were 1.01 (1.00–1.03), 1.13 (1.10–1.16), and 1.23 (1.16–1.31), respectively. After further adjustment for eGFR quintile and hsCRP quintile, the corresponding CAC score ratios in the 1, 2, and ≥3 ECG risk score groups were 1.01 (1.00–1.02), 1.14 (1.10–1.17), and 1.21 (1.13–1.29), respectively.

## 4. Discussion

In this large cohort of 134,802 relatively healthy Korean young adults, we observed a positive association between integrated ECG parameters and the prevalence of CAC scores. In addition, CAC scores progressively increased with higher ECG risk scores, and the association remained significant after adjustment for traditional CVD risk factors and other potential confounders. Our analysis demonstrated that individuals with higher ECG risk scores also had a higher prevalence of a CAC score > 100, for which initiation of statin therapy is strongly recommended [16,17]. Our study highlights that integrated ECG parameters, rather than a single abnormality, were an effective non-invasive tool in identifying individuals at risk for prevalent subclinical atherosclerosis in a low-risk population.

The additive impact of integrating multiple ECG parameters compared with a single parameter and its association with various cardiovascular events have been suggested in previous studies. In a previous study, a higher cumulative ECG risk score was independently associated with a high risk of sudden cardiac death (SCD) in a high-risk population with suspected cardiac arrest. Moreover, the use of an integrated ECG risk score improved the SCD risk stratification compared with the use of patients’ clinical characteristics or severely reduced left ventricular systolic function parameter [9]. The same study showed that the association was attenuated but remained significant even in a healthy general population [9]. Other studies also suggested that combining multiple ECG risk scores was effective in identifying individuals with high SCD risk in the general population [24]. Moreover, a progressive increase in the risk of cardiovascular mortality was associated with an increasing number of major and minor ECG abnormalities [28]. However, studies demonstrating the relationship between the combined ECG risk score and CAC are limited. Only a few have explored the potential of specific abnormal ECG parameters for CAC prediction, resulting in conflicting findings. A previous cross-sectional study of a large multi-ethnic population reported the lack of association between ECG abnormalities and CAC [18]. Another study suggested that ECG abnormalities were associated with CAC burden in an unselected population without known CAD [29]. These data, however, were mostly derived from middle-aged and older individuals, who were generally considered to have elevated CVD risk. The predictive potential of the combined ECG risk score for CAD in young and asymptomatic populations has not been investigated. To the best of our knowledge, our study is the first to demonstrate the potential utility of integrated ECG risk score in estimating the risk of early-stage subclinical atherosclerosis in young adults.

CAC testing is a useful tool for detecting subclinical atherosclerosis [15] and stratifying individuals who may benefit from cholesterol-lowering agents [16]. Although the preventive benefit of therapeutic interventions in at-risk young adults aged 40–49 years has been documented [30], the utility of routine CAC screening in young asymptomatic individuals with a low CVD burden remains unknown. In addition, owing to radiation exposure and cost, its use is not widely applicable in clinical practice. Conversely, ECG is a non-invasive and inexpensive method that is widely available in clinics [6]. In our study, patients with higher ECG risk scores had a higher prevalence of CAC scores > 100, for which initiation of statin therapy is strongly recommended [16,17]. Our study highlights that even in a low-risk population, there are potential clinical gains in leveraging integrated ECG parameters beyond a single abnormality to identify individuals at risk for prevalent significant subclinical atherosclerosis who may benefit from statin therapy. The utility of the integrated ECG score as a screening tool needs to be further assessed in future studies.

The mechanism underlying the association between ECG abnormalities and CAC remains unclear. However, there is some evidence regarding the role of each ECG component composed of ECG risk scores in relation to subclinical and clinical coronary artery disease. T-wave inversion, reflective of ventricular repolarization abnormality and subendocardial ischemia, has been reported to be significantly associated with CAC [12,31]. Prolonged QT interval, an unfavorable prognostic factor of CVD morbidity and mortality, may be a marker for subclinical atherosclerotic disease measured by carotid intima-media thickness [32,33]. Elevated heart rates may indicate autonomic dysfunction and induce damage to the atrial wall, which leads to the development of atherosclerotic disease [34,35]. LVH is also known as a risk factor for CVD, as previous studies have demonstrated a consistently strong association between LVH and CVD [36]. LV wall thickness and concentricity have been associated with CAC and elevated CRP levels, supporting LVH as an independent risk factor for subclinical atherosclerosis [37]. Another study demonstrated that ECG-based evidence of myocardial disease, including borderline LVH, is associated with an elevated CAC burden [29]. In addition, QRS duration has been reported to be an independent predictor of cardiac death and/or nonfatal infarction in patients with suspected coronary artery disease [38]. Taken together, it can be assumed that each individual ECG parameter acts synergistically to represent the incremental risk of CAC via a mechanism that is not fully understood. Hence, further studies are needed to identify the mechanistic link between integrated electrographic changes and CAC.

Our study has several limitations. First, due to the cross-sectional nature of the study, we were unable to determine the causal relationship between exposure and outcome; as a result, there is a possibility of reverse causation or an unclear temporal relationship. Second, we used a self-administered health questionnaire to obtain the medical history, medication history, demographic factors, and lifestyle factors, which may lead to residual and unmeasured confounding factors. Third, the ECG results and CAC scores on MDCT were obtained through a single measurement. However, technicians were trained using the standard protocol, and the results were confirmed by two experienced cardiologists. Fourth, although the locations of inverted T waves can refer to different prognostic values [39], unfortunately the location information was not available in this study. Fifth, we evaluated the association between individual ECG parameters and the prevalence of CAC to determine which individual ECG parameter should be included in ECG risk scoring [24], which has been used in previous studies on the association between integrative ECG scores and sudden cardiac death [9,24]. However, validation studies of this integrative ECG score are not currently available for predicting CAC scores, although coronary artery disease is the most common cause of sudden cardiac death [40]. Similar to other studies on ECG scores, we evaluated the individual ECG parameters in age- and sex-adjusted analysis with respect to the prevalent CAC and then chose ECG parameters that remained independent in the mutually adjusted model. The ECG abnormalities used in the present study have also been reported to be predictors for coronary artery disease events [7,41]. However, further studies are required to confirm and validate this integrative ECG score in predicting CAC. Lastly, our study population predominantly consisted of highly educated, relatively young, and mostly male Korean adults; therefore, the generalizability of our findings to other populations may be limited. Hence, further studies on other races/ethnicities are needed to confirm our findings.

Our study demonstrated a significant association between ECG abnormalities and a higher prevalence of subclinical CAC in a relatively young and low-risk population, with a stronger association being observed with additional abnormal ECG parameters. Our study findings indicate that integrating multiple ECG parameters would help identify individuals who are at an elevated risk of early-stage CVD among low-risk individuals.

## Figures and Tables

**Table 1:** Estimated ^1^ mean value (95%) and adjusted ^1^ proportion (95% CI) of the baseline characteristics of the study population (*n* = 134,802).

Characteristics	ECG Risk Score				*p*-Value
	0 (*N* = 86,702)	1 (*N* = 40,935)	2 (*N* = 6027)	≥3 (*N* = 1138)	
Age (years)	41.3 (41.2–41.3)	41.5 (41.4–41.6)	43.5 (43.4–43.8)	44.2 (43.7–44.7)	<0.001
Male (%)	68.8 (68.5–69.2)	86.9 (86.6–87.2)	86.3 (85.4–87.1)	90.4 (88.7–92.0)	<0.001
Current smoker (%)	23.0 (22.7–23.2)	22.6 (22.3–23.0)	21.5 (20.5–22.5)	21.8 (19.6–23.9)	<0.001
Excess alcohol intake (%) ^2^	16.7 (16.4–17.0)	17.3 (16.9–17.7)	18.9 (17.9–19.8)	21.2 (19.0–23.5)	<0.001
Regular Exercise (%) ^3^	13.8 (13.6–14.0)	13.9 (13.6–14.3)	13.1 (12.2–13.9)	10.3 (8.6–12.1)	<0.001
Highest education level (%) ^4^	83.2 (82.9–83.4)	81.6 (81.3–82.0)	80.0 (79.0–81.0)	79.3 (77.0–81.6)	<0.001
BMI (kg/m^2^) ^5^	24.3 (24.3–24.3)	24.5 (24.5–24.5)	24.7 (24.6–24.8)	25.3 (25.2–25.5)	<0.001
History of HTN (%) ^6^	10.7 (10.5–10.9)	13.7 (13.4–14.1)	19.8 (18.9–20.7)	24.0 (21.7–26.2)	<0.001
Diabetes (%) ^7^	1.3 (1.2–1.4)	1.5 (1.4–1.6)	2.6 (2.2–2.9)	2.9 (2.1–3.7)	<0.001
Family history of heart disease (%)	6.5 (6.4–6.7)	6.5 (6.3–6.8)	5.9 (5.3–6.5)	6.0 (4.6–7.4)	<0.001
Total cholesterol (mg/dL)	199.2 (198.9–199.4)	199.0 (198.7–199.4)	199.9 (199.0–200.8)	201.2 (199.0–203.4)	<0.001
LDL-C (mg/dL)	128.9 (128.7–129.1)	128.7 (128.4–129.0)	128.5 (127.7–129.3)	128.9 (127.7–130.7)	<0.001
HDL-C (mg/dL)	63.7 (63.5–63.9)	63.8 (63.5–64.1)	65.4 (62.6–64.1)	64.3 (62.6–66.0)	<0.001
eGFR < 60 mL/min/1.73 m^2^ (%)	19.6 (19.3–19.9)	19.1 (18.7–19.6)	18.8 (17.7–19.8)	20.7 (18.4–23.1)	0.120
hsCRP (mg/dL)	1.07 (1.05–1.10)	1.26 (1.22–1.29)	1.81 (1.71–1.90)	2.40 (2.17–2.62)	<0.001
CAC score	10.2 (9.6–10.8)	11.6 (10.8–12.5)	23.2 (21.1–25.4)	33.9 (29.0–38.8)	<0.001

^1^ Adjusted for age and sex. ^2^ ≥30 g/day for men and ≥20 g/day for women; ^3^ ≥3 times/week; ^4^ ≥college graduate; ^5^ BMI ≥ 25 kg/m^2^; ^6^ Defined as systolic BP ≥ 140 mmHg, diastolic BP ≥ 90 mmHg, a history of hypertension, or current use of anti-hypertensive medications; ^7^ Defined as a fasting serum glucose level of ≥126 mg/dL, HbA1C of ≥6.5%, a history of diabetes, or current use of anti-diabetic medications. Abbreviations: BMI, body mass index; BP, blood pressure; CAC, coronary artery calcification; CI, confidence interval; eGFR, estimated glomerular filtration rate; HbA1C, hemoglobin A1C; HDL-C, high-density lipoprotein cholesterol; hsCRP, high sensitivity C-reactive protein; HTN, hypertension; LDL-C, low-density lipoprotein cholesterol.

**Table 2:** Analysis of individual ECG parameters with prevalence of CAC.

ECG Parameters	*N* (%) ^1^	ECG Parameters Individually		ECG Parameters in the Same Model	
		PRs (95% CI)	*p* Value	PRs (95% CI)	*p* Value
Heart rate (HR) > 80 bpm	7224 (5.4)	1.18 (1.10–1.27)	<0.001	1.14 (1.06–1.23)	<0.001
PR interval > 220 ms	1689 (1.3)	0.93 (0.81–1.06)	0.775	0.93 (0.81–1.07)	0.783
QRS > 110 ms	37,558 (27.9)	1.08 (1.04–1.12)	<0.001	1.07 (1.03–1.11)	<0.001
Left ventricular hypertrophy (LVH)	3256 (2.4)	1.35 (1.22–1.49)	<0.001	1.30 (1.18–1.44)	<0.001
T wave inversion	462 (0.3)	2.11 (1.70–2.61)	<0.001	1.90 (1.52–2.36)	<0.001
Prolonged QTc	7977 (5.9)	1.22 (1.14–1.30)	<0.001	1.14 (1.07–1.22)	<0.001

^1^ Number of patients with ECG parameters (% of the total study population). The age- and sex-adjusted PRs (95% CIs) for the prevalence of CAC associated with individual ECG parameters were estimated using Poisson regression models with robust variance. Initially, each ECG parameter was analyzed and compared with other ECG parameters in the same model. Abbreviations: CAC, coronary artery calcium; CI, confidence interval; ECG, electrocardiogram; PR, prevalence ratio.

**Table 3:** Prevalence ratios ^1^ (95% CI) for CAC score by ECG risk score in 134,802 study participants.

CAC Score	ECG Risk Score				*p* Value
	0	1	2	≥3	
CAC score = 0	1.00 (base)	1.00 (base)	1.00 (base)	1.00 (base)	
CAC score > 0–100					
Prevalent cases (%)	8461 (9.8)	4901(12.0)	910 (15.1)	197 (17.3)	
Age- and sex-adjusted PR ^1^ (95% CI)	1.00 (reference)	1.09 (1.05–1.14)	1.25 (1.15–1.35)	1.42 (1.20–1.69)	<0.001
Multivariate-adjusted PR ^1^ (95% CI)	1.00 (reference)	1.06 (1.02–1.10)	1.12 (1.03–1.22)	1.19 (1.00–1.42)	<0.001
CAC score > 100					
Prevalent cases (%)	1850 (2.1)	1098 (2.7)	339 (5.6)	85 (7.5)	
Age- and sex-adjusted PR ^1^ (95% CI)	1.00 (reference)	1.08 (1.00–1.18)	1.73 (1.51–1.20)	2.27 (1.74–2.95)	<0.001
Multivariate-adjusted PR ^1^ (95% CI)	1.00 (reference)	1.03 (0.95–1.12)	1.44 (1.25–1.66)	1.75 (1.33–2.29)	<0.001

^1^ Estimated from multinomial logistic regressions, which used CAC scores as outcomes categorized as 0, >0–100, and >100. The multivariate-adjusted model was adjusted for age, sex, center, year of screening, BMI, smoking status, alcohol intake, physical activity, education, history of diabetes, history of hypertension, family history of heart disease, HDL-C, LDL-C, and triglyceride level. Abbreviations: BMI, body mass index; CAC, coronary artery calcium; CI, confidence interval; ECG, electrocardiogram; HDL-C, high-density lipoprotein cholesterol LDL-C, low-density lipoprotein cholesterol; PR, prevalence ratio.

**Table 4:** CAC score ratio ^1^ (95% CI) by ECG risk score in 134,802 study participants.

CAC Score	ECG Risk Score				*p*- Value
	0	1	2	≥3	
Age- and sex-adjusted CAC score ratio (95% CI)	1.0 (reference)	1.03 (1.02–1.04)	1.22 (1.20–1.34)	1.21 (1.18–1.25)	<0.001
Multivariate-adjusted CAC score ratio (95% CI)	1.0 (reference)	1.01 (1.00–1.03)	1.13 (1.10–1.16)	1.23 (1.16–1.31)	<0.001

^1^ Estimated from Tobit regression models using natural log (CAC + 1) as the outcome. The multivariate-adjusted model was adjusted for age, sex, center, year of screening, BMI, smoking status, alcohol intake, physical activity, education, history of diabetes, history of hypertension, family history of heart disease, HDL-C, LDL-C, and triglyceride level. Abbreviations: BMI, body mass index; CAC, coronary artery calcium; CI, confidence interval; ECG, electrocardiogram; HDL-C, high-density lipoprotein cholesterol; LDL-C, low-density lipoprotein cholesterol.

## Data Availability

The data are not available to be shared publicly because we do not have permission from the IRB to distribute the data. However, analytical methods and study materials are available from the corresponding author on reasonable request.
